# Serum brain-derived neurotrophic factor (BDNF) is not regulated by testosterone in transmen

**DOI:** 10.1186/s13293-015-0055-5

**Published:** 2016-01-08

**Authors:** Matthias K. Auer, Rainer Hellweg, Peer Briken, Günter K. Stalla, Guy T’Sjoen, Johannes Fuss

**Affiliations:** Endocrinology, Diabetology and Internal Medicine, Max Planck Institute of Psychiatry, Kraepelinstr. 2-10, 80804 Munich, Germany; Department of Psychiatry, University of Medicine of Berlin, Campus Charité Mitte, Bonhoefferweg 3, 10117 Berlin, Germany; Institute for Sex Research and Forensic Psychiatry, Center for Psychosocial Medicine, University Medical Center Hamburg-Eppendorf, Martininstr. 52, 20246 Hamburg, Germany; Department of Endocrinology, Ghent University Hospital, De Pintelaan 185, 9000 Ghent, Belgium

**Keywords:** Transsexualism, Estradiol, Sex steroids, Testosterone, Brain-derived neurotropic factor, BDNF, Gender dysphoria, Brain morphology, Platelets

## Abstract

Brain morphology significantly differs between the sexes. It has been shown before that some of these differences are attributable to the sex-specific hormonal milieu. Brain-derived neurotrophic factor (BDNF) is involved in myriads of neuroplastic processes and shows a sexual dimorphism. Transsexual persons may serve as a model to study sex steroid-mediated effects on brain plasticity. We have recently demonstrated that serum levels of BDNF are reduced in transwomen following 12 months of cross-sex hormone treatment. We now wanted to look at the effects of testosterone treatment on BDNF in transmen. In contrast to our initial hypothesis, BDNF levels did not significantly change, despite dramatic changes in the sex-hormonal milieu. Our data indicate that testosterone does not seem to play a major role in the regulation of BDNF in females.

**Electronic supplementary material:**

The online version of this article (doi:10.1186/s13293-015-0055-5) contains supplementary material, which is available to authorized users.

## Findings

### Introduction

Men and women show distinct differences with regard to their macroscopic brain architecture though the relevance of these findings on a functional level is far from being elucidated. Progress in magnetic resonance imaging techniques has led to a growing number of studies investigating gender-specific features in brain morphology. Among others, this includes disparities in total volume and white-to-gray-matter ratios as well as regional variances in distinct areas of the brain [1, 2]. While some of these differences seem to have their origin in the prenatal period of development, others first emerge with the onset of puberty [3]. However, sex-specific plasticity of the brain is not restricted to younger individuals. Also in adulthood, drastic changes in the sex-hormonal milieu as seen in androgen deprivation therapy in men with prostate cancer [4] or in endocrine treatment of gender dysphoric individuals are still capable of influencing brain morphology [5]. It is therefore obvious that the shaping of the human brain in a sex-dependent manner is influenced by sex steroids. It has been demonstrated before that sex steroids are directly influencing the neuroplastic potential of the brain such as modulating neurogenesis or neurite outgrowth [6, 7]. In particular, gender dysphoric individuals may serve as a model to study the effects of sex steroids on brain plasticity. Gender dysphoria (GD) is a condition characterized by a strong gender identification that is opposite to the gender an individual was initially assigned to at birth [8]. An etiological reason for GD has not been identified yet, but biological factors have been discussed that might influence gender identity [9–11], as many patients report on GD from the beginning of earliest childhood on [12]. Medical options to overcome the feeling of distress, commonly emerging from the experienced incongruence between physical appearance and gender identification, consist in the initiation of a so-called cross-sex hormone (CSH) treatment and/or gender reassignment surgery. It has been shown before that brain-derived neurotrophic factor (BDNF) levels are reduced in hormonally treated transwomen (male-to-female individuals) in comparison to men from the general population and this has also been suggested to represent an etiological contributor or marker for GD [13]. We could recently demonstrate that 1 year of CSH in transwomen results in a significant drop in peripheral BDNF levels which makes an explanation focusing on the etiology for GD less convincing [14]. This is of certain importance as it has been shown before that BDNF levels are lower in women than in men [15]. As BDNF is involved in a variety of neuroplastic processes such as synaptogenesis, neurogenesis, or myelination [16] and has also been reported to have an impact on macroscopic brain architecture [17, 18], it may therefore play a role in gender-specific and sex steroid-driven differences in brain architecture [19]. BDNF is expressed in a variety of tissues, and BDNF levels in serum are regarded to be closely related to BDNF concentrations in the brain [20] and may provide information on BDNF release and uptake by the central nervous system [21].

Studies investigating the effects of testosterone on BDNF in humans in general, as well as in the setting of CSH in transmen are missing so far. We hypothesized that BDNF levels in transmen would decrease after 1 year of CSH in a well-characterized cohort being part of the European Network for the Investigation of Gender Incongruence (ENIGI), vice versa to the observation made in transwomen. As platelets are the major peripheral source of BDNF and a significant correlation of BDNF with platelet count has been reported [22], but was not assessed in ENIGI, we also investigated in a retrospective analysis within another cohort, if CSH has an effect on platelet characteristics in transmen.

### Material and methods

Participants were part of the ENIGI, a collaboration of four European gender identity clinics (Amsterdam, Ghent, Hamburg, and Oslo) to study the diagnostics and treatment of GD [23]. Subjects included had been exclusively treated at the Department of Endocrinology at the Ghent University Hospital between February 2010 and August 2012. All transmen that participated (*N* = 29) were of Caucasian origin and hormone-naïve at first visit including no use of any hormonal contraceptives at baseline. CSH was initiated with 1000-mg testosterone undecanoate (Nebido®, Bayer) every 12 weeks. All participants gave written informed consent. In addition, we retrospectively evaluated data that were available from the chart files of transmen (*N* = 38) that had been treated at the endocrine outpatient unit of the Max Planck Institute of Psychiatry in Munich (MPIP), Germany, between 2006 and 2014 and of whom data on platelets were available from at least two time-points within an 18-month observation period. Here, all patients had been treatment-naïve at baseline and were subsequently treated with either testosterone enanthate 250 mg every 2–3 weeks or testosterone gel 50 mg per day. The study was approved by the ethical review board of the Ghent University Hospital and the Ludwig Maximilian University in Munich and conducted in accordance with the Declaration of Helsinki.

#### Medical history and examination

Assessment of data on medical history, including comorbidities, mood, and lifestyle parameters such as medication intake, smoking history, and physical exercise as well as a detailed description of the assessments of anthropometry and laboratory measures has been reported before for the ENIGI cohort [24]. Briefly, body weight was measured in light indoor clothing without shoes to the nearest 0.5 kg. Blood drawings took place in the morning between 8:00 and 9:00 a.m. following an overnight fast. After a clotting period of 30–60 min, serum was centrifuged and stored at −80 °C until analysis. 17-β-Estradiol (E2) and testosterone were determined using liquid chromatography tandem mass spectrometry (AB Sciex 5500 triplequadrupole mass spectrometer; AB Sciex, Toronto, Canada) in the ENIGI cohort and by an electrochemiluminescence immunoassay in an automated analyzer (Elecsys 2010; Roche Diagnostics, Mannheim, Germany) in the MPIP cohort. For information on the methods of the determination of follicle-stimulating hormone (FSH), luteinizing hormone (LH), and sex hormone-binding globulin (SHBG) and interassay coefficients of variance (CV), please see [24]. Endogenous levels of BDNF were measured as described previously [22, 25]. Platelets were analyzed by means of low-frequency detection with sheath flow technology using an automated hematologic analyzers (XE 2100®, Sysmex, Kobe, Japan).

Within the ENIGI study group, seven subjects were taking selective serotonin reuptake inhibitors (SSRIs) at baseline due to a positive history of major depression and one was taking haloperidol due to schizophrenia. No subject had started any new psychopharmacological therapy or had been hospitalized for psychiatric reasons, while two patients stopped their medication during follow-up. In the MPI cohort at baseline, two patients were taking SSRIs and one patient a tricyclic antidepressant due to major depression and two subjects took atypical antipsychotics, one due to schizoaffective disorder and another due to bipolar disorder. Of these, one patient terminated intake of SSRIs during the observation period. Patients with a recent history of psychiatric comorbidity and current psychiatric medication intake were excluded in a subanalysis.

#### Statistical analysis

Statistical analysis was carried out using PASW 18.0 (SPSS Inc., Chicago, IL). Data are reported as means (±S.E.M.). For longitudinal comparison, a repeated measure analysis of variance (ANOVA) was used for metric variables and the Wilcoxon signed-rank test or McNemar’s test for ordinal, respectively, dichotomous variables. Significance was evaluated at a probability of 5 % or less.

### Results

As expected, several months of CSH in transmen of the ENIGI as well as of the MPIP sample resulted in a significant change in the sex-hormonal milieu in a cross-sex way (Table 1). However, in contrast to our initial hypothesis, we did not observe any statistically significant effect of the treatment on BDNF levels (*p* = 0.795) (Table 1). This did also not change if excluding those subjects who were taking any psychiatric medication at baseline (data not shown). There was no significant effect of CSH on platelet count (*p* = 0.952) or volume (*p* = 0.663) in the Munich cohort. The transmen from Ghent and the cohort from Munich did not significantly differ at baseline with regard to BMI (24.2 kg/m^2^ ± 1.0 vs. 23.6 kg/m^2^ ± 0.8, *p* = 0.435), age (29.1 years ± 1.1 vs. 27.2 years ± 1.1; *p* = 0.069) and did not include significantly more or less active smokers (24.1 vs. 15.8 %, *p* = 0.075).

**Table 1:** BDNF, general characteristics, thrombocyte measures

	Baseline		After 12 months				
	Mean	S.E.M.	Mean		S.E.M.		*p*
ENIGI transmen							
General characteristics							
Age (years)	29.1	1.8					
BMI (kg/m^2^)	24.2	1.0	25.2		0.9		*0.016*
Weight (kg)	66.5	3.1	69.8		3.0		*0.007*
Waist (cm)	77.4	2.6	79.8		2.5		n.s.
Laboratory measures							
BDNF (pg/ml)	5635.1	291.0	5573.4		302.2		n.s.
FSH (U/L)	6.2	1.5	4.5		2.1		n.s.
LH (U/L)	8.3	1.6	10.4		4.8		n.s.
Estradiol (pg/mL)	116.5	14.5	51.6		8.7		*0.002*
Testosterone total (ng/dL)	42.1	8.4	653.3		44.2		*<0.001*
Testosterone free (nmol/L)	0.4	0.1	13.6		1.2		*<0.001*
SHBG (nmol/L)	78.0	8.9	36.2		2.7		*<0.001*
Lifestyle							
Sports index	2.9	0.9	2.8		0.9		n.s.
Work index	2.8	0.9	3.0		0.9		n.s.
Freetime index	2.8	0.9	3.0		0.9		n.s.
	*N*	%	*N*		%		
Current smoking	7	24.1	3		10.2		n.s.
	Baseline		3–7 months		8–18 months		
	Mean	S.E.M.	Mean	S.E.M.	Mean	S.E.M.	
MPIP transmen							
General characteristics							
Age (years)	27.2	0.6					
BMI (kg/m^2^)	23.6	0.8	24.8	0.8	24.9	0.8	*<0.001*
Laboratory measures							
FSH (U/L)	6.3	0.6	5.8	0.9	12.8	5.3	n.s.
LH (U/L)	12.2	2.2	5.9	1.5	9.8	3.3	*0.01*
Estradiol (pg/mL)	113.2	17.5	129.3	42.8	66.0	11.2	*0.022*
Testosterone total (nmol/L)	3.0	0.8	24.3	12.8	2.5	3.2	*<0.001*
Lifestyle							
Current smoking	*N*	%	*N*	%	*N*	%	
Yes	6	15.8	5	13.2	4	10.5	n.s
No	32	84.2	28	73.7	28	73.7	n.s.
Not documented			5	13.2	6	15.8	n.s
Thrombocyte measures	Mean	S.E.M.	Mean	S.E.M.	Mean	S.E.M.	
Thrombocyte count (10^9/l)	270.3	11.4	270.1	11.5	277.6	26.3	n.s.
Mean thrombocyte volume (fl)	10.3	0.2	10.4	0. 2	10.4	0.2	n.s.

*ENIGI* European Network for the Investigation of Gender Incongruence, *BDNF* brain-derived neurotrophic factor, *MPIP* Max Planck Institute of Psychiatry

Twelve months of CSH resulted in significant changes in sex hormones in both cohorts. In contrast to our initial hypothesis, we did however not observe any statistically significant effect of the treatment on BDNF levels in transmen

P-values in italic indicate a significant difference

### Discussion

In the present study, we demonstrate that 12 months of cross-sex hormone treatment do not significantly affect serum BDNF levels in transmen. This was against our initial hypothesis as we had expected that BDNF levels would rise vice versa to the decrease in transwomen we and others had observed before [13, 14].

Men in general seem to have higher peripheral levels of BDNF than women, and it has been suggested that the differences in platelet content/uptake of BDNF between men and women may explain this observation, though differences in BMI are also a potential confounder in this regard [15].

That sex steroids do play a role in peripheral BDNF regulation in natal females is supported by the fact that women with amenorrhea as well as postmenopausal women have decreased BDNF levels and hormone replacement therapy results in restoration up to levels seen in fertile women [26]. Cubeddu and colleagues reported that BDNF is increased in the second half of the menstrual cycle [27]. With regard to sex steroids, the second—luteal phase—of the menstrual cycle is primarily characterized by the presence of progesterone [28] but also by an increase in androgen secretion in comparison to the follicular phase including a significant peak at midcycle [29]. That progesterone is a potential confounder is supported by the fact that progesterone is at least capable of increasing central BDNF release, potentially thereby mediating some of its suggested neuroprotective effects [30]. Progesterone levels were not available from the ENIGI study, and though levels of LH, FSH, and estradiol allow a rough estimate of the cycle phase, we cannot rule out that differences in cycle phase between the subjects at baseline have influenced the results of our study.

Our findings speak against a role of peripheral BDNF for the observed increase in brain volume in transmen following CSH [31, 32]. Only few studies have investigated the direct effects of testosterone on BDNF levels, and there is in particular a paucity of studies that have been performed in human subjects. Rodent studies indicate that testosterone withdrawal in males subsequent to gonadectomy decreases BDNF within the hippocampus [33] as well as in certain motoneurons [34]. One study did report on increased BDNF levels in women suffering from polycystic ovary syndrome (PCOS), which is characterized among others by the presence of hyperandrogenemia [35]. However, in this study, this finding was attributed to an increase of BDNF release from the ovaries as BDNF was also significantly elevated in the follicular fluid of these women.

The fact that CSH in transmen results in a decrease in BDNF levels, while BDNF remains unaffected by testosterone treatment in transwomen, could suggest that other mechanisms interfere with peripheral BDNF release that might mask the effects of testosterone on BDNF in transmen. Though platelets were not measured in ENIGI, we did not observe any alterations in platelet count or volume in transmen in a comparable patient and treatment setting, making an interfering effect of this potential confounder unlikely. As this does not exclude the possibility that the reported decrease in BDNF in transwomen was mediated by a decrease in platelet counts in these patients, we also took a look on transwomen that had been treated at the MPIP with regard to platelet characteristic (Additional file 1: Table S1). Interestingly, while platelet counts were not affected by CSH in transmen, we observed a small, albeit significant increase in platelet counts in transwomen. This indicates that estradiol affects thrombocyte formation or release and is therefore in line with the fact that platelet counts are higher in women compared to men [36].

It speaks however against a direct effect of total platelet counts on the earlier observed reduction in BDNF levels in transwomen. Increased platelet counts should otherwise have resulted in an increase and not a decrease of serum BDNF [10]. Yet, CSH may alter BDNF levels by directly affecting the BDNF release from platelets. To our knowledge, there is no study available that has investigated the direct effects of sex steroids on BDNF release in vitro. However, testosterone has shown to induce platelet activation [37], and this in turn has been associated with BDNF release [38].

Although it is suggested that platelets are the major peripheral source of BDNF, we can however not exclude that counteracting effects with regard to peripheral BDNF release are present in transmen involving ovarian BDNF release. In addition to its presence within the follicular fluid, it has been shown that BDNF is also increased in menstrual blood and also expressed within cells of the endometrium [39]. As CSH in transmen results in amenorrhea and therefore ovarian arrest, as well as atrophy of the endometrium, a decrease in BDNF would be the logical consequence. Further studies could therefore, e.g., in particular focus on the effects of the treatment on BDNF levels in ovarian and endometrial tissue which would be available for study purposes as these sexual organs are usually removed during sex reassignment surgery. It has also been demonstrated before that peripheral BDNF levels are lower in depressed subjects in comparison to healthy controls and that they can be increased following introduction of antidepressant treatment [40, 41]. In this context, BDNF has also been suggested as potential predictor for remission [40]. The exact mechanism for this finding has not been completely elucidated so far, but it seems that this effect is independent of the administered drug class [40, 41]. Though platelets express serotonin and noradrenalin receptors and are thereby directly targeted by antidepressant drugs such as SSRI and noradrenalin reuptake inhibitors (NARI) [42], there is also evidence that peripheral BDNF levels do respond to non-pharmacological antidepressant treatments as well [43]. Though mood disorders are common in transsexual individuals [44], we did not observe any changes in our results if we excluded those patients that had been pharmacologically treated due to any psychiatric comorbidity. We thus doubt that alteration in BDNF levels as observed earlier in transwomen reflects a particular mood status, as it has been repeatedly reported that there is an improvement in the general well-being and depressive symptoms in transmen as well as in transwomen [45, 46], following initiation of CSH, in particular during the first year of medical treatment.

Our study results nonetheless challenge the idea that at least peripheral BDNF levels mediate the macroscopic brain changes in transmen, observed during CSH, and rather support the notion that direct effects of testosterone are responsible for this finding.

We can only speculate if BDNF in this particular setting plays any role in cognitive functioning. There are only few studies that have investigated the effects of CSH on cognition in gender dysphoric individuals. CSH in transwomen seems to have little effects on memory function [47] though it may, e.g., affect activation patters in fMRI paradigms including mental rotation tasks [48]. To date, it is not known, whether this effect is directly attributable to macroscopic changes in brain structure or a result of altered neuronal activation patterns [49].

In conclusion, our data do not indicate that testosterone does play a major role in the regulation of peripheral BDNF in females. However, we cannot exclude that potential interfering mechanisms, we could not account for in the present study setting, may have influenced the results.

## Additional file

Additional file 1: Table S1. General characteristics and platelet measures in transwomen. (DOCX 18.5 kb)

## Abbreviations

BDNF: brain-derived neurotrophic factor
CSH: cross-sex hormone
CV: coefficients of variance
E2: 17-β-estradiol
ENIGI: European Network for the Investigation of Gender Incongruence
FSH: follicle-stimulating hormone
GD: gender dysphoria
LH: luteinizing hormone
MPIP: Max Planck Institute of Psychiatry in Munich
PCOS: polycystic ovary syndrome
SHBG: sex hormone-binding globulin
SSRI: selective serotonin reuptake inhibitor
