# Supplementary material for: Serum brain-derived neurotrophic factor (BDNF) is not regulated by testosterone in transmen
Source: Biol Sex Differ. 2016 Jan 8;7:1. doi: 10.1186/s13293-015-0055-5 (PMC4705590; doi:10.1186/s13293-015-0055-5)
Supplement: Additional file 1: Table S1. — General characteristics and platelet measures in transwomen. (DOCX 18.5 kb) [file 13293_2015_55_MOESM1_ESM.docx]

We retrospectively evaluated data that were available from the chart files of transwomen (N = 42) that had been treated at the endocrine outpatient unit of the Max-Planck Institute of Psychiatry in Munich (MPIP), Germany, between 2006-2014 and of whom data on platelets were available from at least two time-points within a 18 months observation period. All patients had been treatment-naïve at baseline and were subsequently treated with 2-4 mg 17β-estradiol either given orally or as transdermal gel application.

Here, in contrast to transmen, we did observe a small but significant increase in platelet counts in transwomen during the first 18 months of hormonal treatment (p = 0.008) (Table S1).

**Table S1. General characteristics and platelet measures in transwomen**

| **MPIP** | | | | | | | |
| --- | --- | --- | --- | --- | --- | --- | --- |
|  | **Baseline** | | **3-7 months** | | **8-18 months** | |  |
| **General characteristics** | **Mean** | **S.E.M** | **Mean** | **S.E.M** | **Mean** | **S.E.M** | **p** |
| Age [years] | 36.0 | 12.8 |  |  |  |  |  |
| BMI [kg/m²] | 24.9 | 1.3 | 24.9 | 1.2 | 25.2 | 1.1 | n.s. |
| **Laboratory measures** |  |  |  |  |  |  |  |
|  |  |  |  |  |  |  |  |
|  |  |  |  |  |  |  |  |
| FSH [U/L] | 9.1 | 3.5 | 0.8 | 0.3 | 5.1 | 2.6 | **0.031** |
| LH [U/L] | 6.7 | 1.8 | 1.1 | 0.5 | 3.9 | 2.0 | n.s. |
| Estradiol [pg/mL] | 29.7 | 2.2 | 352.2 | 83.1 | 169.5 | 19.7 | **<0.001** |
| Testosterone total [nmol/l] | 18.3 | 6.5 | 1.5 | 2.0 | 1.9 | 2.2 | **<0.001** |
| **Lifestyle** |  |  |  |  |  |  |  |
| Current smoking | **N** | **%** | **N** | **%** | **N** | **%** |  |
| yes | 13.0 | 31.0 |  |  |  |  |  |
| no | 28.0 | 66.7 |  |  |  |  |  |
| Not documented | 1.0 | 2.4 |  |  |  |  |  |
|  |  |  |  |  |  |  |  |
| **Thrombocyte measures** | **Mean** | **S.E.M** | **Mean** | **S.E.M** | **Mean** | **S.E.M** |  |
|  |  |  |  |  |  |  |  |
| Thrombocytes count [10^9/l] | 248.6 | 9.8 | 264.1 | 10.1 | 265.0 | 9.7 | **0.008** |
|  |  |  |  |  |  |  |  |
| Mean thrombocytes volume [fl] | 10.0 | 0.3 | 9.8 | 0.3 | 10.1 | 0.2 | n.s. |
|  |  |  |  |  |  |  |  |
